# The Shifting Climate Portfolio of the Greater Yellowstone Area

**DOI:** 10.1371/journal.pone.0145060

**Published:** 2015-12-16

**Authors:** Adam J. Sepulveda, Michael T. Tercek, Robert Al-Chokhachy, Andrew M. Ray, David P. Thoma, Blake R. Hossack, Gregory T. Pederson, Ann W. Rodman, Tom Olliff

**Affiliations:** 1 US Geological Survey, Northern Rocky Mountain Science Center, 2327 University Way, Suite 2, Bozeman, MT, 59715, United States of America; 2 Walking Shadow Ecology, Gardiner, MT, 59030, United States of America; 3 National Park Service, Greater Yellowstone Inventory and Monitory Network, 2327 University Way, Suite 2, Bozeman, MT, 59715, United States of America; 4 US Geological Survey, Aldo Leopold Wilderness Research Institute, 790 E. Beckwith Avenue, Missoula, MT, 59801, United States of America; 5 National Park Service, Yellowstone Center for Resources, PO Box 168, Yellowstone NP, WY, 82190, United States of America; 6 National Park Service, Intermountain Region Landscape Conservation and Climate Change Division, 2327 University Way, Suite 2, Bozeman, MT, 59715, United States of America; Universidade de Vigo, SPAIN

## Abstract

Knowledge of climatic variability at small spatial extents (< 50 km) is needed to assess vulnerabilities of biological reserves to climate change. We used empirical and modeled weather station data to test if climate change has increased the synchrony of surface air temperatures among 50 sites within the Greater Yellowstone Area (GYA) of the interior western United States. This important biological reserve is the largest protected area in the Lower 48 states and provides critical habitat for some of the world’s most iconic wildlife. We focused our analyses on temporal shifts and shape changes in the annual distributions of seasonal minimum and maximum air temperatures among valley-bottom and higher elevation sites from 1948–2012. We documented consistent patterns of warming since 1948 at all 50 sites, with the most pronounced changes occurring during the Winter and Summer when minimum and maximum temperature distributions increased. These shifts indicate more hot temperatures and less cold temperatures would be expected across the GYA. Though the shifting statistical distributions indicate warming, little change in the shape of the temperature distributions across sites since 1948 suggest the GYA has maintained a diverse portfolio of temperatures within a year. Spatial heterogeneity in temperatures is likely maintained by the GYA’s physiographic complexity and its large size, which encompasses multiple climate zones that respond differently to synoptic drivers. Having a diverse portfolio of temperatures may help biological reserves spread the extinction risk posed by climate change.

## Introduction

Climate is a major factor driving the ecosystem processes that affect the distribution, interactions, phenology and life-history of species [1, 2]. Rapid changes in climate over the past 50 years have altered species distributions and food-web structure, induced phenological mismatch, and increased extinction risk for at least 20% of the world’s species [3]. Consequently, climate change may surpass habitat destruction as the greatest global threat to biodiversity [4], prompting the need for a better understanding of how climate variability is changing at small spatial extents (< 50 km), especially in important biological reserves. At these smaller extents, the ecological effects of a changing climate are manifest and managed.

Over the past two decades climate change research has largely focused on changes in mean meteorological conditions over large extents [5]; indeed, climate change is epitomized by an increase in the global mean annual temperature [6]. However, there is growing awareness that local meteorological variability within seasons occurring at critical times relative to important biophysical processes is a dominant force structuring ecological communities [5, 7, 8]. The mean condition is merely the central tendency of a distribution of variable conditions, including extremes, and it is ultimately this variability that drives local adaptations [5, 8]. The influence of anomalous climatic events is exemplified by the shift in average beak depth of Darwin’s finches in the Galapagos in response to a severe drought, rather than to a long-term decline in precipitation. Climatic variability, particularly extreme events, has already had important effects on the population and community dynamics of a variety of animal and plant species [9, 10]. It is widely projected that as the planet warms, the shape and location of the central tendency of meteorological distributions at smaller spatial scales will change [11].

Physiographic features on the earth’s surface, such as topography, can modulate meteorological conditions experienced at smaller spatial extents, such that neighboring sites with different aspects or elevations have different temperature distributions [12–14]. However, the physiographic controls on local climates may be overwhelmed by the increasing strength of climate change [15, 16]. For example, local temperature isoclines have increased in elevation as global temperatures have warmed, which can result in a positive snow albedo feedback that leads to further warming over a larger area and reductions in late Spring snow [16–19]. Similarly, long-term changes in synoptic conditions could decrease the frequency of cold-air pooling in valley bottoms [18]. For reasons such as these, the spatial extent of extreme weather events have increased [17, 20–22]. For example, temperatures exceeding 3 standard deviations of the 1951–1980 average covered < 0.1% of the global land area from 1951–1980 but now occur over 10% of global land area [20]. Thus, it is possible that climate change has not only increased climate variability within a location, but also increased the spatial synchrony of temperatures among locations regardless of topographic heterogeneity.

Surprisingly, there has been little consideration of how meteorological distributions have changed among nearby sites, even though this extent (< 50 km) is needed to understand ecological responses to shifts in climate within biological reserves. Positive spatial autocorrelation of meteorological variables, such as temperature, can be particularly detrimental when species dynamics are synchronized across space and multiple populations are exposed to extreme climatic events [23–25]. Alternatively, we also know that fine-scale features, like soil moisture and topography, can modify the intensity of climate events and create high spatial variability in meteorological conditions [14, 16]. The resulting spatial variability may help lower the extinction risk posed by climate change [25–27], similar to how spatial patchiness increases the resilience of landscapes to disturbance [28]. However, we know little about how climate varies among nearby sites because most climate studies occur at the global [3], national [20] or regional extent (e.g., western U.S. [13]). Clearly, a better understanding of climatic variability at smaller spatial extents will enhance our understanding of how future climatic changes may influence populations, communities and ecosystems in biological reserves.

**Fig 1:**
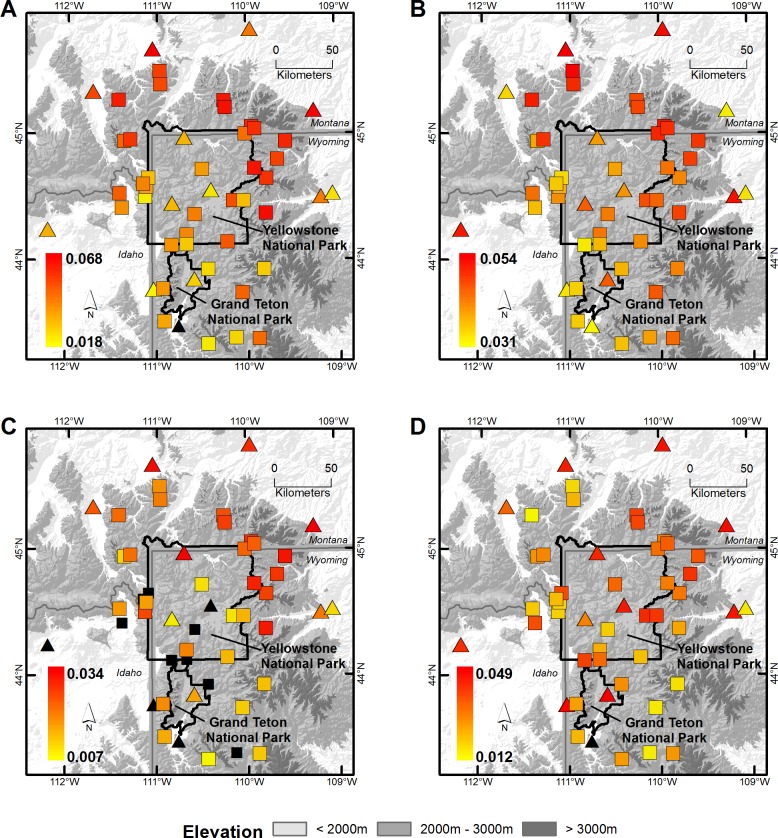
Location of weather station sites and annual trends in minimum and maximum temperatures for winter and summer. Annual trends in the mean minimum (A, C) and maximum (B, D) temperatures for the winter (A, B) and summer (C, D) at SNOTEL (□) and COOP weather station sites (∆) in the Greater Yellowstone Area using the modeled SNOTEL + COOP data, 1948–2012. All sites had positive trends and trend magnitude is indicated in the legend with warmer colors.

Here, we test if the shape of the distributions of temperature conditions among nearby sites has shifted or altered from 1948–2012 in the Greater Yellowstone Area (GYA) of the interior western United States (Fig 1). The GYA, which includes Yellowstone and Grand Teton national parks, encompasses ~ 9 million hectares, is one of the last largely intact temperate ecosystems on Earth, and is the largest protected area in the Lower 48 states [29]. With vast tracts of public lands, the topographically variable GYA encompasses multiple climatic regions [13, 14] and offers a range of habitat refugia for species, including mountain ranges, volcanic plateaus and intermontane basins. The protected regions within the GYA also serve as the headwaters of three major rivers in the central and western United States: the Snake-Columbia, the Green-Colorado, and the Yellowstone-Missouri rivers. The GYA has experienced substantial shifts in seasonal and annual temperatures, reduced snowpack, accelerated melt and glacial recession as a result of rapid global warming [30, 31]. We focused on temperature because it has a ubiquitous influence on biological systems and historical temperature data are readily available across the GYA. However, temperature is only one of many variables that comprise the GYA’s climate portfolio. Understanding how climate change may be altering the temperature portion of the climate portfolio in the GYA is urgent given projected increases in regional temperatures [32] and the importance of this area in providing critical habitat for some of the world’s most iconic wildlife, such as grizzly bears, bison, wolves and cutthroat trout as well as important ecosystem services to downstream water users. Furthermore, improving our understanding of the observed changes in the climate portfolio can be used in developing robust mitigation and adaptation strategies [33].

## Materials and Methods

### Approach

The GYA climate portfolio consists of sites found in a transition zone between the Southwest and Pacific Northwest precipitation response to the El Niño-Southern Oscillation. High-elevation snow basins and sites near the western GYA show a Pacific Northwest-type response and have different seasonal patterns of precipitation than lower elevations and sites near the eastern GYA, which show a southwestern-type response [14]. Within each climate regime, sites occur across an elevation gradient that spans valley-bottoms at 522 m to mountain peaks at 4,206 m. To capture the variability and changes in temperature across this large area and elevation gradient, we used empirical data from snowpack telemetry (SNOTEL) and Cooperative Observer Network (COOP) weather stations and modeled weather data [34, 35]. These data can be accessed at http://dx.doi.org/10.6084/m9.figshare.1615873.

Empirical and modeled datasets were used because no individual meteorological dataset is without systematic and random bias. For example, SNOTEL and COOP stations provide high-frequency, year-round temperature data, which closely represent the microclimates experienced by biota. In the GYA, there is a dense network of SNOTEL stations at higher elevations (generally above 2000 m) and COOP stations at lower elevations (1200–2400 m) at varying aspects and slopes, so these empirical data do provide important information about the variety of meteorological conditions that occur in this region (Fig 1 and S1 Table). However, SNOTEL and COOP stations are not distributed evenly across the landscape, especially at the highest elevation zones (> 2900 m). SNOTEL stations have limited temporal coverage since a majority of SNOTEL stations only started to record temperature in the early 1990s, while COOP stations have a large number of missing data points from different time periods. Station data are also sensitive to different microclimate effects and have non-climactic temperature jumps and trends resulting from changes in observation protocols, instrumentation or station siting [35, 36].

The modeled data set is from Oyler et al. [35] and interpolates SNOTEL and COOP minimum (T_min_) and maximum (T_max_) daily temperatures from 1948–2012. This data set has no missing values and input station data are homogenized using the GHCN/USHCN Pairwise Homogenization Algorithm [37], which adjusts for numerous issues including time of day bias, time of observations and time series difference comparisons to nearby valley stations. These adjustments make the modeled data set more suitable for long-term climate trend analyses [34]. However, biases in this dataset also exist because the homogenization algorithm corrects for artificial changes only in the daily mean, which may smooth out extreme temperatures. Additionally, valley station data and elevation-based predictors are used to homogenize and infill missing values, which may result in additional problems due to the challenges of modeling climate in complex terrain. While imperfect, these modeled data do allow us to assess temperature distributions over greater time than station data alone. Further, our use of multiple lines of evidence, in this case station data and modeled data, will strengthen conclusions.

To describe changes in the GYA climate portfolio, we focused our analyses on temporal shifts and shape changes in the annual distributions of T_min_ and T_max_ air temperatures among SNOTEL and COOP station sites. We calculated distributions based on the following daily data sets: (1) modeled SNOTEL and COOP data from 1948–2012, (2) empirical SNOTEL data from 1990–2012, (3) modeled SNOTEL data from 1990–2012, (4) modeled SNOTEL and COOP data from 1990–2012, and (5) modeled SNOTEL data from 1948–2012. Our primary interest is data set 1, but data sets 2–5 are needed to understand how the modeled data affect results and to identify the relative contribution of high vs. low elevation sites to the climate portfolio (see S1 Fig for results and discussion). We chose not to include empirical COOP station data in comparisons because the large number of missing data points from different time periods did not allow us to assess empirical distributions.

### Analyses

We included SNOTEL stations in the GYA with less than 5 days of missing temperature each month. This limited us to 37 stations (S1 Table) with empirical and modeled station data from 1990–2012 and modeled station data from 1948–2012. We constrained COOP stations in the GYA to USHCN sites or those that spanned 1948–2012 with less than 15% of missing values. This limited us to 13 stations with modeled station data from 1990–2012 and 1948–2012. To minimize spatial autocorrelation, we did not include additional COOP stations with a greater number of missing values since neighboring stations are used to infill missing data. For all included empirical stations and modeled station data, we calculated average seasonal T_min_ and T_max,_ where Summer is July–September, Fall is October–December, Winter is January–March, and Spring is April–June [38]. For COOP stations with missing values, we only calculated average monthly temperatures for stations with less than 5 days of missing data each month and with less than 15 days missing each year. To test if our characterization of seasons affected results, we also calculated average monthly T_min_ and T_max_ using modeled SNOTEL and COOP data from 1948–2012 (data set 1).

To thoroughly assess changes in the GYA temperature portfolio, we considered multiple metrics describing the seasonal distributions of temperature regimes for each year across the data sets’ period of record. First, we considered changes in the location of a distribution’s central tendency by quantifying the mean value and the 25^th^, 50^th^ and 75^th^ percentiles of each distribution; increases in these metrics over time indicate that distributions have shifted towards a warmer climate. Second, we considered changes in the distribution’s shape through measures of variance, skewness (i.e., asymmetry of the distribution about its mean) and kurtosis (i.e., the height and sharpness of the peak relative to the rest of the data) of each distribution. Together, shifts and shape changes provide information about changes in climate variability and the spatial synchrony of temperatures among sites [9, 11]. Third, we estimated annual Moran’s I from all stations to test if spatial autocorrelation of seasonal T_min_ and T_max_ has increased with time. Moran’s I varies from -1 (dispersed) to +1 (clustered), with values near zero indicating a random spatial pattern [39].

Next, we quantified within station changes in the annual distributions of temperature to identify spatial patterns and the specific regions within the GYA that have demonstrated higher rates of change. Here, we used modeled SNOTEL and COOP station data from 1948–2012 (data set 1) to quantify distributional changes within each station. Similar to our among-station comparisons, we assessed within-station changes in seasonal T_min_ and T_max_ distributions by evaluating trends in the 25^th^, 50^th^ and 75^th^ percentiles and mean, variance, skewness and kurtosis.

We used the different metrics to quantify how seasonal T_min_ and T_max_ distributions have changed both among- and within-stations. We used the non-parametric Theil-Sen test to calculate the slopes of these descriptive statistics, including Moran’s I, as a function of time and the Mann-Kendall test to test if the slopes were significantly different than zero. The Theil-Sen test provides a more robust slope estimate than the least-squares method, as the influence of outliers or extreme values in the time series effect is minimized [40, 41]. We used α = 0.10 as a significance threshold for all statistical analyses. To visually describe shifts and shape changes, we binned annual summaries by decade and present these decadal summaries of seasonal T_min_ and T_max_ distributions. To assess if among-station trends were driven by few or many SNOTEL station locations, we determined the proportion of stations with significant slopes. Finally, we tested for correlations using Pearson correlation coefficients (*r*) between station-specific changes in temperature metrics and elevation, latitude and longitude. Elevation, latitude and longitude were not collinear (*r* < 0.23, *P* > 0.11).

## Results

### Among-site

#### Data set 1 (Modeled SNOTEL + COOP data, 1948–2012; n = 50)

**Fig 2:**
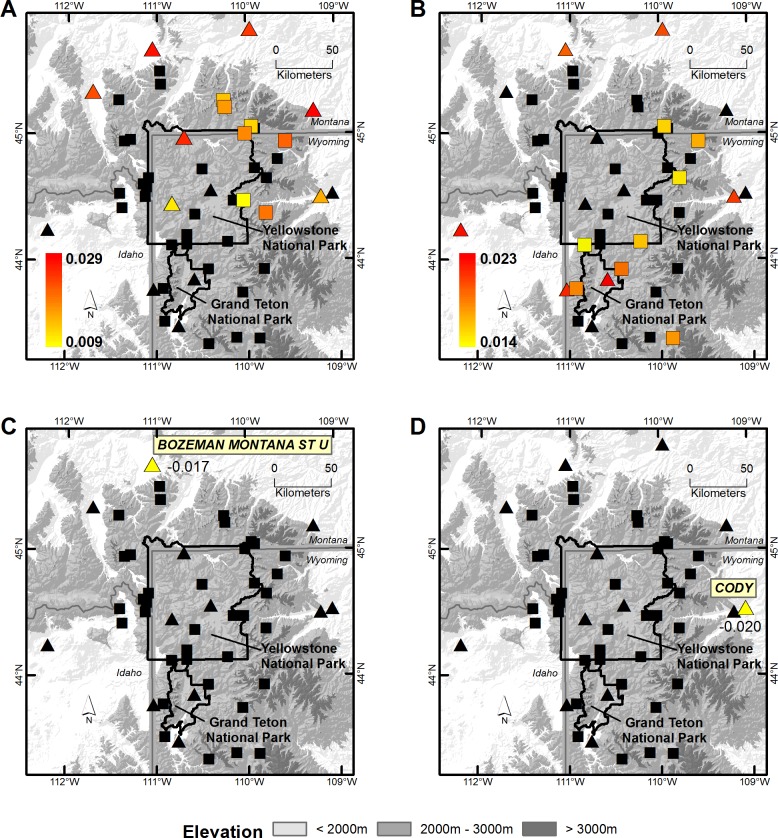
Annual trends in minimum and maximum temperatures for Spring and Fall. Trends in the mean minimum (A, C) and maximum (B, D) temperatures for the Spring (A, B) and Fall (C, D) at SNOTEL (□) and COOP weather station sites (∆) in the Greater Yellowstone Area using the modeled SNOTEL + COOP data, 1948–2012. Trend magnitude is indicated in the legend with warmer colors indicating larger trends. Sites where the trend did not differ from zero are filled in black. For Fall, the trend estimate and site name is indicated adjacent to the sites filled in yellow.

**Fig 3:**
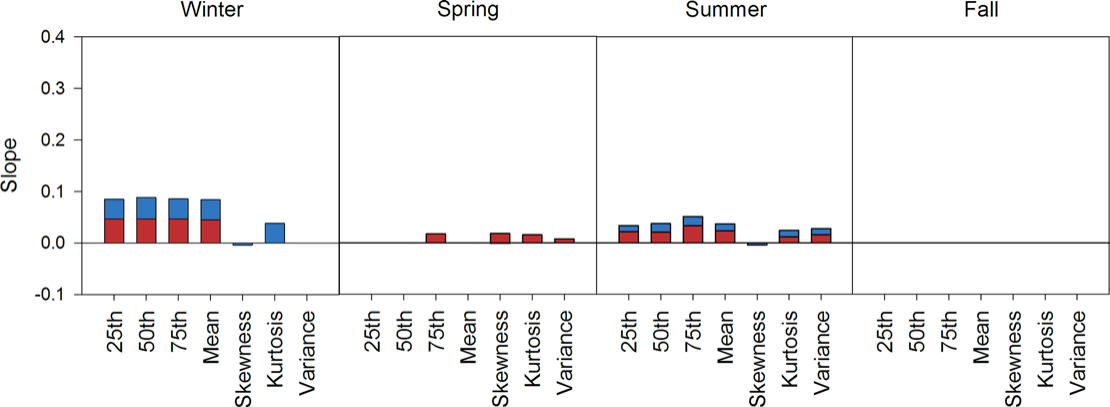
Trends of the descriptive statistics for seasonal minimum (blue) and maximum (red) temperature distributions using the modeled SNOTEL + COOP data, 1948–2012. Only slopes that were significantly different from zero are plotted; missing values indicate non-significance.

T_min_ and T_max_ distributions shifted to the right (warmer temperatures) in the Winter and Summer (Figs 1–3). For Winter and Summer T_min_ and T_max_ distributions, the slopes of the mean and 25^th^ to 75^th^ percentile were significantly greater than zero (S2 Table). In the Winter, the mean and interquartile range (25^th^ to 75^th^ percentile) of the T_min_ and T_max_ distributions increased by 0.25–0.26°C and 0.29–0.32°C per decade from 1948–2012. In the Summer, the mean and interquartile range (25^th^ to 75^th^ percentile) of the T_min_ and T_max_ distributions increased by 0.09–0.13°C and 0.15–0.22°C per decade. In the Spring, only the slope of the 75^th^ percentile of the T_max_ distribution was positive, indicating the greatest changes occurred at those stations with relatively warm temperatures. No distributions metrics were significant in the Fall.

Changes in the shape of the distributions of T_min_ and T_max_ were apparent, but slopes associated with shape changes were much smaller than slopes associated with shifts (Fig 3, S2 Table). Slopes of skewness were significant and less than zero for only T_min_ distributions in Winter, Spring, and Summer, though they only decreased by less than -0.03°C per decade. A decrease in skewness indicates that the mass of the distribution is shifting towards warmer temperatures. Slopes of kurtosis were significantly greater than zero for T_min_ distributions in Winter and Summer and significantly less than zero for T_max_ distributions in Summer. Kurtosis of the T_min_ Winter and Summer distributions increased by 0.25 and 0.09°C per decade, which indicates increases in the central and extreme values during these seasons. Kurtosis of the T_max_ Summer distribution decreased by -0.02°C per decade, which indicates that this distribution has flattened. Slopes of variance were significantly greater than zero for T_min_ distributions in the Summer and T_max_ distributions in the Spring and Summer, which indicates a lengthening of the right tail with record hot weather and a majority of stations that are warmer than the mean. Variance of the Summer T_min_ distribution increased by 0.10°C per decade, while variance of the Spring and Summer T_max_ distributions increased by 0.06 and 0.10°C per decade. Trend estimates for the slope and intercept for seasonal T_min_ and T_max_ distribution statistics are presented in S2 Table.

The spatial distributions of seasonal T_min_ and T_max_ remained near-random across the period of record. Moran’s I ranged from 0–0.12 across all years and seasons, which indicates that temperatures at nearby stations were only minimally more related than temperatures at distant stations. Trends in Moran’s I were only significant (*p* < 0.05) for Winter T_min_, Summer T_min_ and T_max_, and Fall T_max_; but these trends were near zero (-0.0001–0.0004), which indicates little change in spatial autocorrelation over time.

**Fig 4:**
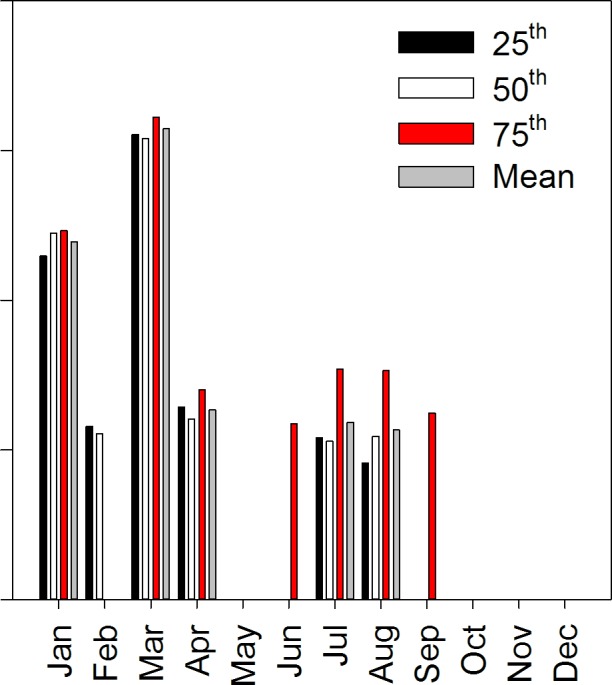
Trends of the descriptive statistics for monthly minimum (A, C) and maximum (B, D) temperature distributions using the modeled SNOTEL + COOP data, 1948–2012. Top panels (A, B) show slopes for statistics that describe distribution shifts and bottom panels (C,D) show slopes for statistics that describe distribution shape changes. Only slopes that were significantly different from zero are plotted; missing values indicate non-significance.

For monthly analyses, T_min_ and T_max_ distribution shifts were largest in March and then January (Fig 4). Smaller T_min_ and T_max_ distribution shifts also occurred in April, July and August. Slopes for skewness, kurtosis and variance did not differ greatly among months. Trend estimates for the slope and intercept for monthly T_min_ and T_max_ distribution statistics are presented in S3 Table.

#### Data sets 2–5

Detailed results for these data sets are presented in S1 Fig. In general, modeled data provided a more conservative estimate of distributional shifts and shape changes than empirical SNOTEL data, but empirical and modeled data had similar seasonal patterns (Fig 3 and S1 Fig). The period of record did affect result interpretations. Directional shifts toward increasing temperatures in the distributions of T_min_ and T_max_ were large during Fall 1990–2012, but were not different from zero in Fall 1948–2012 (Fig 3 and S1 Fig). Winter and Summer distributions of T_min_ and T_max_ from 1948–2012 shifted to the right, but were seldom different from zero in modeled data from 1990–2012 (Fig 3 and S1 Fig).

### Within-site

#### Modeled SNOTEL + COOP data, 1948–2012

**Fig 5:**
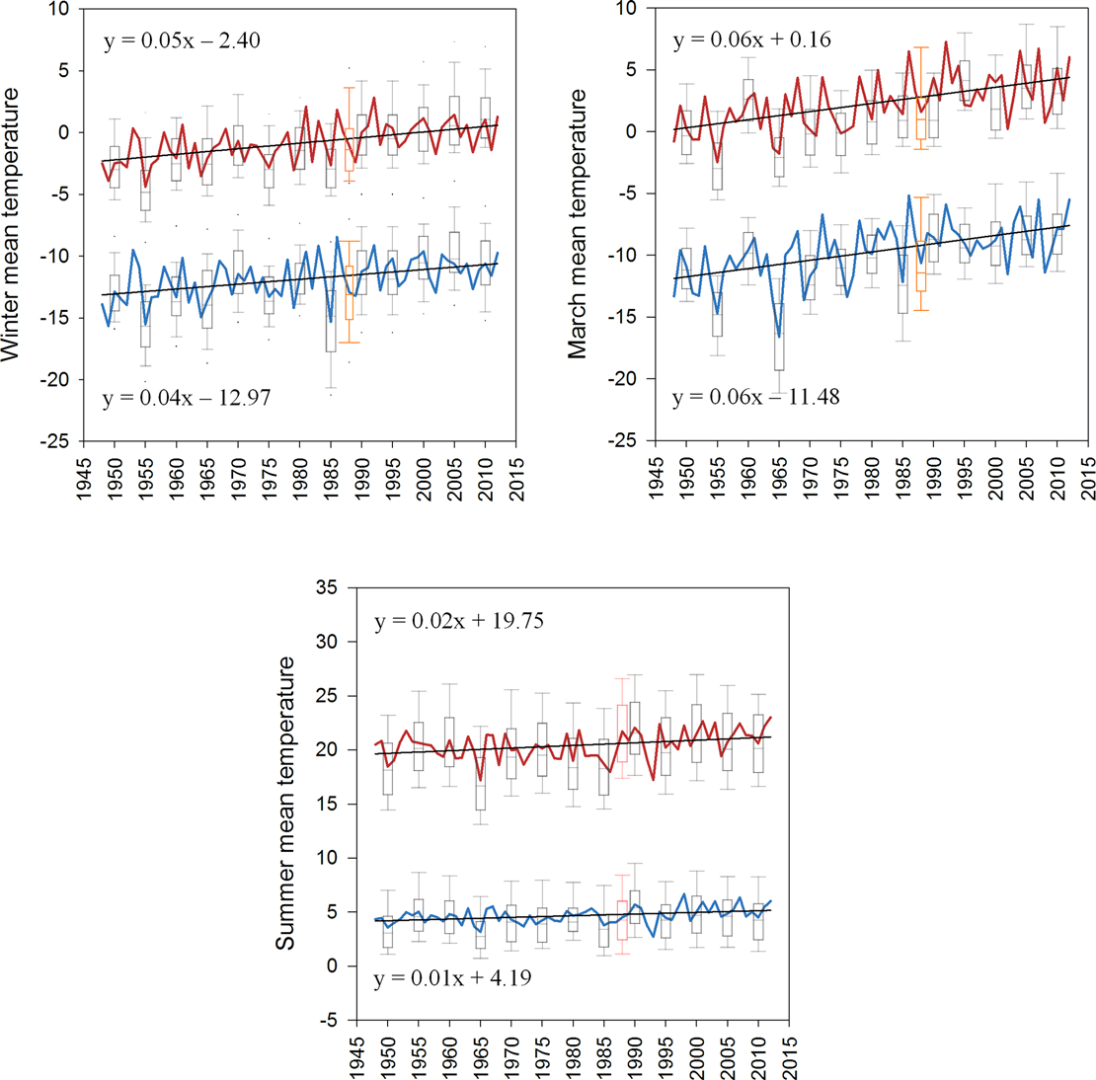
Time series of Winter, March and Summer maximum (red) and minimum (blue) temperatures using the modeled SNOTEL + COOP data, 1948–2012. Trend lines and their equations for each time series are shown and are positive and significant (*p < 0*.05; non-parametric Theil-Sen estimator and Mann-Kendall test). Box plots show the median value, box boundaries indicate the 25^th^/75^th^ percentiles and whiskers indicate the 5^th^/95^th^ percentiles of annual temperature values for 50 stations at 5-year intervals and for 1988 (orange), the year of the large Yellowstone fires.

T_min_ and T_max_ distributions shifted towards warmer Winter and Summer climates in all 50 stations (Fig 1, Fig 5, S2 Fig). In the Winter, these shifts were most pronounced in the northern region of the GYA; slopes associated with T_min_ and T_max_ shifts increased with latitude (*r* = 0.37–0.63, *P* = < 0.04). In the Summer, these shifts were pronounced in the northeast region of the GYA and at low elevation stations across the region; slopes associated with T_min_ shifts increased with latitude and longitude (*r* = 0.41–0.50, *P* = < 0.01), while slopes associated with T_max_ shifts decreased with elevation (*r* = -0.33 –-0.54, *P* = < 0.02). Fewer than 13 stations in the Spring and 7 stations in the Fall demonstrated shifts in distribution during this period (Fig 2).

Similar to our among-station analyses, T_min_ and T_max_ distribution shape changes were less common than shifts. The Winter T_min_ distribution was the only season where shape changes occurred at a majority of stations. Kurtosis significantly increased at 52% of sites and increased with latitude (*r* = 0.43, *P* = 0.03), which indicates that stations in the northern region had an increase in central and extreme values. Variance significantly increased at 88% of sites and increased with elevation (*r* = 0.36, *P* = 0.02). Because slopes of the 25^th^ and 75^th^ percentiles were also positive, the positive variance slope indicates that high elevation stations had an increase in extremely warm weather. In other seasons, slopes associated with T_min_ and T_max_ distribution shape changes were significantly different from zero at less than 42% of sites.

## Discussion

Many studies have projected or identified recent increases in global mean surface temperatures, but local-to-regional changes in temperature can be more varied because of the interactions of local, synoptic, and anthropogenic drivers [22, 42]. Understanding how temperature regimes have changed at sub-grid scales is critical because the ecological effects of a changing climate are manifest and managed at these smaller extents. In the GYA, which provides refuge to some of the world’s most iconic wildlife, we documented consistent patterns of warming since 1948 with the most pronounced seasonal changes occurring during the Winter (JFM) and the most pronounced monthly changes occurring in March. Warming in March across all elevations below 3749 m has also been observed in the Colorado Rocky Mountains [43]. Mean Winter T_min_ and T_max_ from modeled data increased by 0.26 and 0.32°C per decade from 1948–2012 in the GYA (Fig 5, S2 Table), which is comparable to Winter warming rates (0.32°C per decade during 1955–1996) in the Tibetan plateau[44], but larger than the 0.18°C and 0.14°C per decade increase in the global average surface temperature and average surface temperatures observed for North American mountains, respectively, over comparable periods of record [18, 45]. In addition, the distributions of Winter and Summer temperatures in the GYA proceeding 1990 have been similar to and occasionally warmer than the distributions of Winter and Summer temperatures in 1988—the year of the large Yellowstone fires that had historically hot and dry conditions (Fig 5; [28]). Given our analyses and that of Pederson et al. [38], the greater Rocky Mountain region, including the GYA, has experienced rapid changes in temperature. Moreover, temperatures in the GYA are projected to increase ~4.5–5.5°C by 2099 [32].

**Fig 6:**
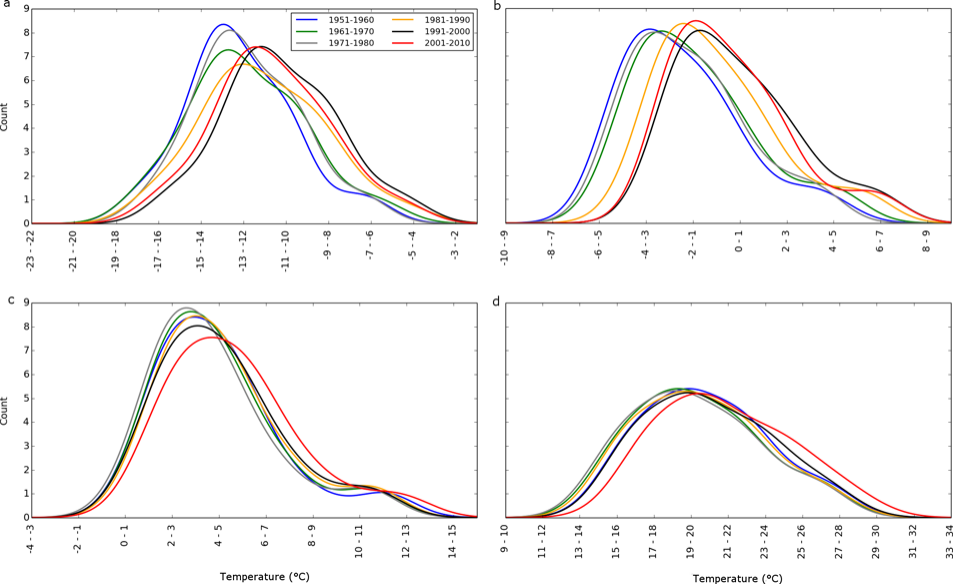
Decadal distributions of minimum (a, c) and maximum (b, d) Winter (a, b) and Summer (c, d) temperatures using the modeled SNOTEL + COOP data, 1950–2010. Distributions were smoothed using kernel density estimators.

Though temperatures warmed at all sites, little change in the shape of the temperature distributions across sites since 1948 suggests the GYA maintains a diverse annual portfolio of temperatures (Fig 6). Annually, the spatial variation in temperatures is likely maintained by the GYA’s physiographic complexity and its large size, which encompasses multiple climate zones that respond differently to synoptic drivers [13, 14]. Protection of an area that has high spatial variability in temperatures was unintentional–the GYA’s extent was first defined as the area necessary to sustain the Yellowstone grizzly bear population [46]. Nevertheless, the bear’s large range was fortuitous because the spatial variation in temperatures may foster adaptive potential to warming temperatures for many GYA organisms, adding another dimension to the umbrella species concept [47]. Adaptive potential is a function of genetic variation and intraspecific genetic variation can evolve in response to habitat heterogeneity [48]. Genetic variation across space fosters the adaptedness of populations as environmental conditions, like air temperature, change [49]. Therefore, protecting areas with spatial variation in environmental conditions may provide a strategy for increasing species’ resilience to rapid climate change [25, 33, 50, 51]. Arguably, our results support the importance of large or highly connected reserves that contain a diversity of environmental conditions as a climate adaptation strategy to reduce extinction risk and foster resilience [25, 33, 50]. This mosaic of environmental conditions echoes the landscape-level patch dynamic concept of fires in the GYA, where a mosaic of stands of different age classes reduces the flammability of the landscape [28].

The concept of spreading ecological risk across multiple species, populations, life-histories or habitats has been termed the *portfolio effect*, similar to the risk-reducing effect and economic value of diversifying financial assets [27, 52]. Central to this economic theory is that risk-reduction is accomplished by selecting assets that change in value in dissimilar ways, such that a collection of investment assets has collectively lower risk than an individual asset [53]. If sites in our GYA climate portfolio analysis are considered assets and temperatures are considered their value, than the GYA climate portfolio is not without risk despite limited evidence for increased spatial synchrony in temperatures. Sites did not converge on similar temperatures over time, but they did respond to a changing climate in a similar way–the mean and 25–75^th^ percentiles of T_min_ and T_max_ distributions shifted towards warmer Winter and Summer climates in all 50 sites (Fig 1 and Fig 5). However, sites had different rates of temperature change so temperature-driven extinction risk across the entire GYA is still lower than extinction risk at any one site. For example, Winter T_min_ and T_max_ change over time increased with latitude, such that sites across all elevations in the Gallatin and Beartooth-Absorka Mountains in the northern portion of the GYA warmed at a faster rate than sites further south (Figs 1 and 2). As long as warming temperatures remain within species’ thermal tolerance limits at some sites (e.g., microrefugia [42]) or species are able to disperse to more suitable habitats, this portfolio of temperature diversity may help to spread the extinction risk posed by a changing climate. This latter point underscores the importance of identifying how the spatial arrangement of the GYA climate portfolio influences dispersal for less vagile species. On the contrary, continued temperature shifts that reduce the proportion or arrangement of sites with suitable thermal regimes (i.e., assets) will ultimately compromise the overall portfolio of the GYA. Other values of the GYA’s climate portfolio, like precipitation and soil moisture, that also influence the distribution and abundance of biota may have very different spatial patterns than temperature and require additional evaluation.

### Empirical and modeled data

Seasonal trends in temperature were influenced by the data set and period of record (S1 Fig). Modeled data from Oyler et al. [35] that corrected for inhomogeneities reduced the magnitude of significant seasonal trends relative to the uncorrected, empirical data for the 1990–2012 period of record. This modeled data also provided a more conservative estimate of warming at higher elevation SNOTEL stations and, as a consequence of the homogenization algorithm that attempts to correct for artificial increases in SNOTEL temperature data [34, 35], we found inconsistent support for elevation-dependent warming. Comparisons of 1948–2012 to 1990–2012 modeled temperature data indicate that the period of record determined seasons with significant trends. For example, warming of T_min_ and T_max_ distributions was large during Fall (OND) 1990–2012, but was not different from zero in Fall 1948–2012 (Fig 3 and S1 Fig). Others have also found that the Fall has warmed at a faster rate than other seasons in recent decades compared to 1948–2010, where Fall was defined as September—November [43, 54]. Identifying the potential biases of empirical and modeled data sets are important when developing climate adaptation and management strategies because societal and ecological implications of Winter warming in a snow-driven system like the GYA are likely to be different than implications of Fall warming.

## Conclusions and Implications

We documented increases in Winter and Summer temperatures across all sites in the GYA. The warming Winter temperatures in the GYA are of large concern because the majority of surface water in this region originates as mountain snowpack. These surface waters feed three major rivers that provide critical societal and environmental services: the Snake-Columbia, the Green-Colorado, and the Yellowstone-Missouri rivers. Increases in Winter and Spring temperatures in the West result in less snow accumulation in the Winter and earlier timing of water released from the snowpack [38, 55–57], which affect the timing of water delivery to downstream irrigation users, municipalities, and hydropower production facilities and influence recreational water uses (e.g., angling and boating) in gateway and downstream communities [58]. Importantly, we documented that much of the Winter temperature warming has occurred in March, which is the primary snow-producing month in this region [59]. With median March T_min_ approaching 0°C and median March T_max_ now much greater than 0°C, the fraction of precipitation that falls as rain rather than snow has likely increased. The large influence of March on Winter warming trends also underscores how aggregations of the data to seasons can influence interpretation of important meteorological trends.

Temperature has a ubiquitous influence on physical and biological systems. For example, the interactions of warming Winter temperatures, which influence the total accumulation and melt-out timing of the snowpack, have a cascading influence on the rate in-stream flows warm and are lost to evapotranspiration, the desiccation of soils and vegetation, and consequently fire dynamics and resources available to wildlife [60]. As such, climate change impacts have already been documented in the GYA (e.g., trout [61] and whitebark pine [62]). The warming temperatures in the GYA present significant challenges for managing ecological integrity and the maintenance of biodiversity for two reasons. First, the core of this region is comprised of federally protected areas, including designated wilderness, where active management and intervention are controversial [63]. Second, some of the species that are most sensitive to warming temperatures (e.g., boreal toad [*Anaxyrus boreas*]) may not receive equitable management attention relative to the GYA’s iconic megafauna (S4 Table; [64]). While this type of conservation bias is not uncommon [65], our results suggest that improved understanding of the spatial heterogeneity and arrangement of temperatures and other components of the climate portfolio within the GYA may help manage transformation to a warmer GYA with minimal change to the character of the GYA.

## Supporting Information

S1 Fig Results and discussion for data sets 2–5. (PDF)

S2 Fig The proportion of sites in each season with significant trends for each descriptive statistic for minimum and maximum temperature distributions using the modeled SNOTEL + COOP data, 1948–2012. (PDF)

S1 Table Description of snowpack telemetry (SNOTEL) and Cooperative Observer Network (COOP) weather stations used in this study. (PDF)

S2 Table Slopes and intercepts for seasonal T_min_ and T_max_ distribution metrics using the modeled SNOTEL + COOP data, 1948–2012. (PDF)

S3 Table Slopes and intercepts for monthly T_min_ and T_max_ distribution metrics using the modeled SNOTEL + COOP data, 1948–2012. (PDF)

S4 Table Management prioritization of species relative to their climate change sensitivity. (PDF)
