# Supplementary material for: The Shifting Climate Portfolio of the Greater Yellowstone Area
Source: PLoS One. 2015 Dec 16;10(12):e0145060. doi: 10.1371/journal.pone.0145060 (PMC4681470; doi:10.1371/journal.pone.0145060)
Supplement: S1 Fig — (PDF) [file pone.0145060.s001.pdf]

S1 Figure

Data sets 2 -5: Modeled data provided a more conservative estimate of distributional shifts and shape changes than empirical SNOTEL data (Figure S4). Slope values for all T_{min} and T_{max} distribution metrics from dataset 2 (empirical SNOTEL data, 1990 - 2012) were much larger than slope values from dataset 3 (modeled SNOTEL data, 1990 – 2012). In dataset 2, T_{min} distributions shifted to the right in all seasons and T_{max} distributions shifted to the right in the Winter, Summer and Fall. Shifts in T_{min} and T_{max} were greatest in the Fall. In dataset 3 (modeled SNOTEL data, 1990 – 2012), T_{min} distribution shifts only occurred in the Fall and T_{max} distribution shifts only occurred in the Summer and Spring. Slope values associated with these shifts were smaller than in dataset 2.

Seasons with distributional shifts did not change when modeled COOP data were included. For the 1990 – 2012 period in datasets 3 and 4, T_{min} shifted in the Fall and T_{max} shifted in the Summer and Fall (Fig. 4). T_{min} and T_{max} shifts were largest in the Fall. For the 1948 – 2012 period in datasets 1 and 5, T_{min} shifted in the Winter and Summer and T_{max} shifted in the Winter, Spring and Summer (Fig. 2 and Figure S4). Shifts in T_{min} and T_{max} were largest in the Winter.

Inclusion of modeled COOP data did alter the shape of T_{min} and T_{max} distributions, but slope values associated with shape changes were still lower than slope values associated with shifts. For 1990 – 2012 T_{min} distributions, inclusion of modeled COOP data increased variance slopes in the Spring and Summer and decreased the variance slope in the Fall (Figure S4). For 1990 – 2012 T_{max} distributions, the 25th percentile slope in the Summer was greater than zero for modeled SNOTEL data, while the 75th percentile slope in the Summer was greater than zero when modeled COOP data were included (Figure S4). Similarly for 1948 – 2012 T_{max} distributions, the 25th percentile slope in the Spring was greater than zero for modeled SNOTEL data, while the 75th percentile slope, skewness, kurtosis and variance in the Spring were greater when modeled COOP data were included (Fig. 2 and Figure S4). Taken together,

these results indicate that the coolest SNOTEL sites have become warmer, while the warmest and lower elevation COOP sites have become even warmer.

Finally, comparisons of 1948 – 2012 and 1990 – 2012 T_{min} and T_{max} distributions indicate that the period of record affects result interpretations. Rightward distribution shifts in T_{min} and T_{max} were large during Fall 1990 – 2012, but were not different from zero in Fall 1948 – 2012 (Figure S4). Winter and Summer 1948 – 2012 T_{min} and T_{max} distributions shifted to the right, but 1990 – 2012 modeled data seldom differed from zero (Fig. 2 and Figure S4).

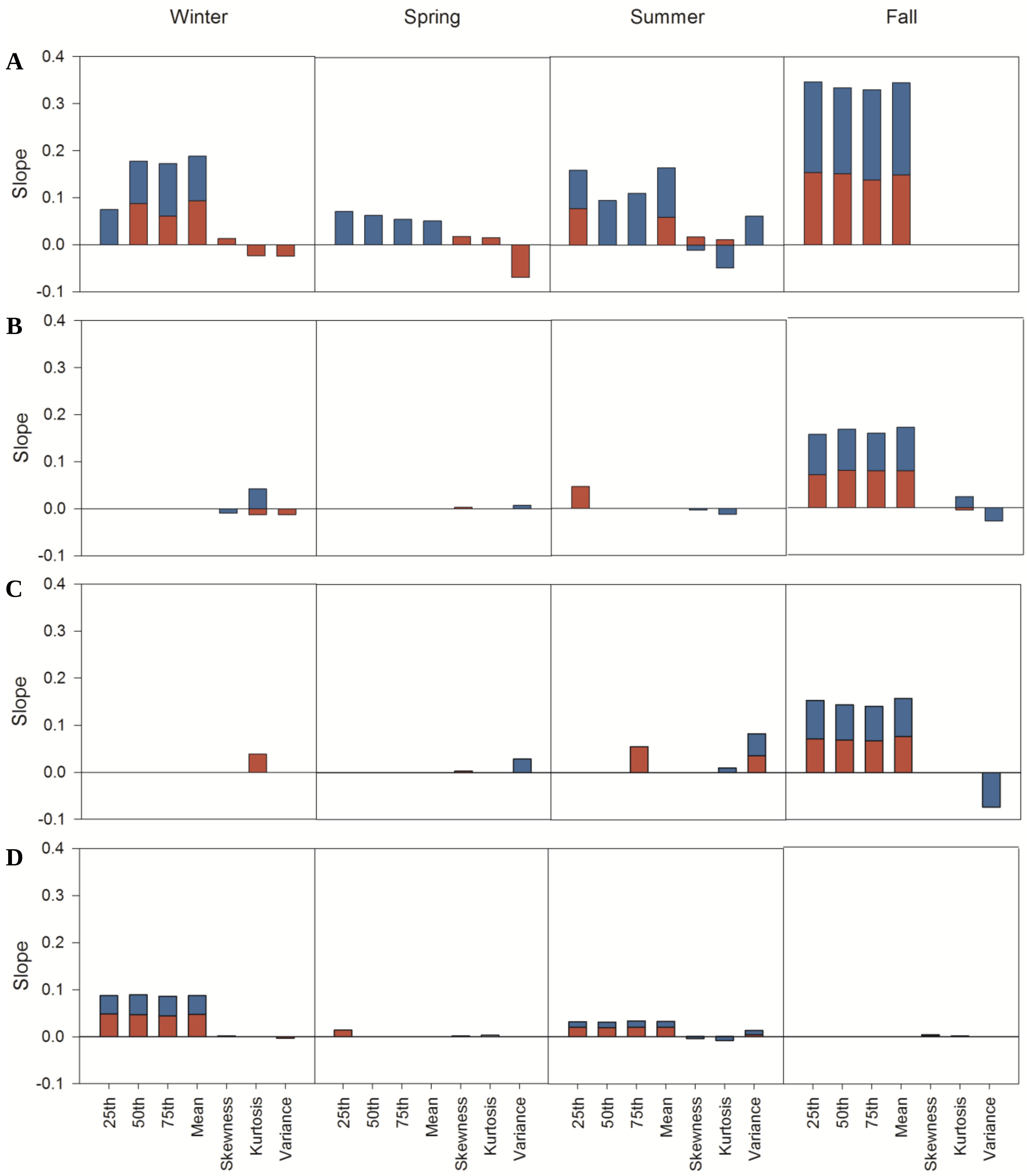

S4 Figure. Slope of the trend of the descriptive statistics for seasonal minimum (blue) and maximum (red) temperature distributions using (A) SNOTEL data, 1990 – 2012, (B) modeled SNOTEL data, 1990 – 2012, (C) modeled SNOTEL + COOP data 1990 – 2012, and (D) modeled SNOTEL data 1948 – 2012. Only slopes that were significantly different from zero are plotted; missing values indicate non-significance.
