# Supplementary material for: The Shifting Climate Portfolio of the Greater Yellowstone Area
Source: PLoS One. 2015 Dec 16;10(12):e0145060. doi: 10.1371/journal.pone.0145060 (PMC4681470; doi:10.1371/journal.pone.0145060)

S2 Figure. Proportion of sites in each season with significant trends (*P < 0.10*) for each descriptive statistic for minimum (blue) and maximum (red) temperature distributions using the modeled SNOTEL + COOP data, 1948 – 2012.

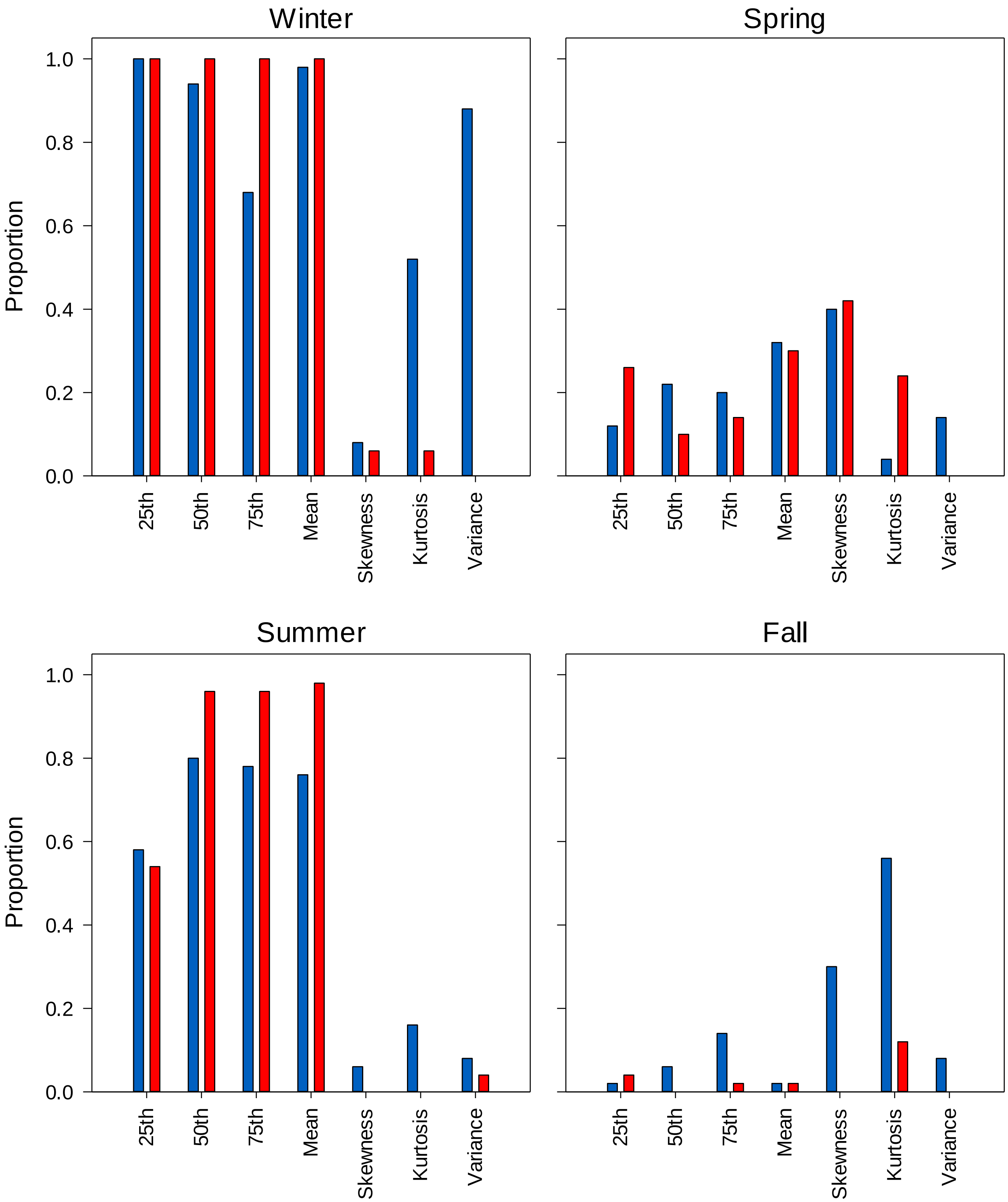

Supplement: S2 Fig — (PDF) [file pone.0145060.s002.pdf]
