# Supplementary material for: The Shifting Climate Portfolio of the Greater Yellowstone Area
Source: PLoS One. 2015 Dec 16;10(12):e0145060. doi: 10.1371/journal.pone.0145060 (PMC4681470; doi:10.1371/journal.pone.0145060)
Supplement: S1 Table — (PDF) [file pone.0145060.s003.pdf]

S1Table

S1 Table. Type (snowpack telemetry [SNOTEL] or Cooperative Observer Network [COOP]), geospatial location, elevation, and aspect of weather stations used in this study.

**Station**	**Type**	**Latitude**	**Longitude**	**Elevation (m)**	**Aspect (°)**
Alta1 NNW, WY	COOP	43.7727	-111.034	1962	313
Big Timber, MT	COOP	45.832	-109.95	1250	352
Bozeman, MT	COOP	45.6784	-111.047	1472	4
Buffalo Bill Dam, WY	COOP	44.5017	-109.183	1638	92
Cody, WY	COOP	44.5147	-109.045	1552	2
Dubois, ID	COOP	44.2436	-112.201	1669	253
Ennis, MT	COOP	45.3394	-111.711	1511	331
Jackson, WY	COOP	43.4866	-110.761	1910	10
Lake Yellowstone, WY	COOP	44.5619	-110.399	2399	101
Moran, WY	COOP	43.8566	-110.589	2072	17
Old Faithful, WY	COOP	44.4569	-110.833	2243	295
Red Lodge, MT	COOP	45.1863	-109.247	1698	70
Mammoth, WY	COOP	44.9766	-110.696	1920	108
Base Camp, WY	SNOTEL	43.9333	-110.433	2143	203
Beartooth Lake, MT	SNOTEL	44.7833	-109.567	2853	359
Beaver Creek, MT	SNOTEL	44.95	-111.35	2393	230
Black Bear, MT	SNOTEL	44.5	-111.117	2490	43
Blackwater, WY	SNOTEL	44.3833	-109.8	2981	25
Box Canyon, MT	SNOTEL	45.2833	-110.25	2033	331
Canyon, WY	SNOTEL	44.7167	-110.533	2399	135
Carrot Basin, MT	SNOTEL	44.9667	-111.283	2743	125
Evening Star, WY	SNOTEL	44.65	-109.783	2804	358
Fisher Creek, MT	SNOTEL	45.0667	-109.95	2774	53
Grand Targhee, ID	SNOTEL	43.7833	-110.933	2822	268
Granite Creek, WY	SNOTEL	43.35	-110.433	2063	280
Grassy Lake, WY	SNOTEL	44.1333	-110.833	2214	156
Gros Ventre Summit, WY	SNOTEL	43.3833	-110.133	2267	26
Gunsight Pass, WY	SNOTEL	43.3833	-109.867	2993	244
Island Park, ID	SNOTEL	44.4189	-111.371	1917	147
Lewis Lake Divide, WY	SNOTEL	44.2	-110.667	2393	135
Lick Creek, MT	SNOTEL	45.5	-110.967	2091	338
Lone Mountain, MT	SNOTEL	45.2833	-111.433	2707	200
Madison Plateau, MT	SNOTEL	44.5833	-111.117	2362	354
Monument Peak, MT	SNOTEL	45.2167	-110.233	2698	336
Northeast Entrance, MT	SNOTEL	45.0056	-110.014	2240	192
Parker Peak, WY	SNOTEL	44.7333	-109.917	2865	111
Phillips Bench, WY	SNOTEL	43.5167	-110.917	2499	135
Shower Falls, MT	SNOTEL	45.4	-110.95	2469	42
Snake River Station, WY	SNOTEL	44.1333	-110.667	2109	47

Sylvan Lake, WY	SNOTEL	44.4833	-110.15	2566	241
Sylvan Road, WY	SNOTEL	44.4667	-110.033	2170	159
Thumb Divide, WY	SNOTEL	44.3667	-110.567	2432	21
Togwotee Pass, WY	SNOTEL	43.75	-110.05	2920	109
Two Ocean Plateau, WY	SNOTEL	44.15	-110.217	2816	352
West Yellowstone, MT	SNOTEL	44.65	-111.1	2042	3
Whiskey Creek, MT	SNOTEL	44.6108	-111.15	2073	300
White Elephant, WY	SNOTEL	44.5333	-111.417	2350	28
White Mill, WY	SNOTEL	45.05	-109.9	2652	37
Wolverine, WY	SNOTEL	44.8	-109.65	2332	97
Younts Peak, WY	SNOTEL	43.9333	-109.817	2545	5
