# Supplementary material for: The Shifting Climate Portfolio of the Greater Yellowstone Area
Source: PLoS One. 2015 Dec 16;10(12):e0145060. doi: 10.1371/journal.pone.0145060 (PMC4681470; doi:10.1371/journal.pone.0145060)
Supplement: S2 Table — (PDF) [file pone.0145060.s004.pdf]

S2 Table: Slopes and intercepts for seasonal T_{min} and T_{max} distribution metrics using the modeled SNOTEL + COOP data, 1948 – 2012. We used the non-parametric Theil-Sen estimator to calculate the slopes of these descriptive metrics as a function of time and the Mann-Kendall test to test if the slopes were significantly different than zero.

		T_{min}			T_{max}		
Season	Metric	Slope	Intercept	p	Slope	Intercept	p
Winter	25th	0.038	-14.750	0.001	0.047	-4.391	0.000
	50th	0.041	-13.519	0.000	0.047	-2.888	0.000
	75th	0.039	-11.369	0.000	0.047	-1.061	0.000
	Mean	0.039	-12.969	0.000	0.046	-2.400	0.000
	Skewness	-0.004	0.435	0.021	0.002	0.799	0.182
	Kurtosis	0.038	-13.787	0.000	0.004	0.169	0.193
	Variance	0.011	6.285	0.336	-0.002	6.182	0.991
Spring	25th	0.004	-2.427	0.353	0.008	9.653	0.248
	50th	0.005	-1.464	0.239	0.008	11.509	0.272
	75th	0.006	0.002	0.287	0.017	13.762	0.083
	Mean	0.005	-0.930	0.244	0.011	11.985	0.129
	Skewness	-0.001	1.203	0.089	0.000	0.561	0.314
	Kurtosis	0.005	-1.565	0.226	-0.001	-0.406	0.319
	Variance	0.003	5.153	0.217	0.008	8.948	0.105
Summer	25th	0.012	2.405	0.006	0.022	17.206	0.015
	50th	0.016	3.746	0.001	0.021	19.386	0.006
	75th	0.018	5.255	0.001	0.033	21.669	0.000
	Mean	0.013	4.192	0.002	0.024	19.745	0.006
	Skewness	-0.004	1.161	0.000	-0.001	0.390	0.178
	Kurtosis	0.012	3.538	0.002	-0.003	-0.494	0.000
	Variance	0.012	6.057	0.007	0.016	10.231	0.000
Fall	25th	0.000	-9.483	0.734	-0.005	0.894	0.626
	50th	-0.001	-7.970	0.883	-0.007	2.578	0.571
	75th	0.000	-6.236	0.839	-0.001	4.476	0.973
	Mean	0.000	-7.684	0.751	-0.005	2.947	0.734
	Skewness	0.000	0.602	0.892	0.000	0.774	0.476
	Kurtosis	0.000	-8.382	0.803	-0.002	-0.034	0.144
	Variance	-0.001	4.961	0.768	0.001	6.646	0.919
