# Supplementary material for: The Shifting Climate Portfolio of the Greater Yellowstone Area
Source: PLoS One. 2015 Dec 16;10(12):e0145060. doi: 10.1371/journal.pone.0145060 (PMC4681470; doi:10.1371/journal.pone.0145060)
Supplement: S3 Table — (PDF) [file pone.0145060.s005.pdf]

S3 Table: Slopes and intercepts for monthly T_{min} and T_{max} distribution metrics using the modeled SNOTEL + COOP data, 1948 – 2012. We used the non-parametric Theil-Sen estimator to calculate the slopes of these descriptive metrics as a function of time (i.e., trend) and the Mann-Kendall test to test if the slopes were significantly different than zero. Only metrics with *p <0.11* are shown.

		T_{min}			T_{max}		
**Month**	**Metric**	**Slope**	**Intercept**	***p***	**Slope**	**Intercept**	***p***
January	25th	0.042	-16.917	0.019	0.046	-7.074	0.001
	50th	0.044	-14.835	0.008	0.049	-5.825	0.000
	75th	0.046	-12.973	0.006	0.049	-4.008	0.001
	Mean	0.043	-14.706	0.016	0.048	-5.378	0.001
	Skewness				0.004	0.900	0.105
February	25th				0.023	-4.005	0.083
	50th				0.022	-2.479	0.098
	Skewness	-0.006	0.450	0.003			
	Kurtosis	-0.008	0.292	0.012			
March	25th	0.063	-13.169	0.000	0.062	-1.654	0.000
	50th	0.063	-11.991	0.000	0.062	-0.119	0.000
	75th	0.055	-9.650	0.000	0.064	1.755	0.000
	Mean	0.060	-11.484	0.000	0.063	0.161	0.000
	Skewness				0.002	0.708	0.101
April	25th	0.015	-7.252	0.062	0.026	3.895	0.028
	50th	0.018	-5.983	0.039	0.024	5.588	0.053
	75th	0.013	-4.324	0.085	0.028	7.658	0.034
	Mean	0.016	-5.575	0.068	0.025	6.255	0.037
May	Skewness	-0.002	1.224	0.085	0.001	0.506	0.051
	Kurtosis	-0.010	1.428	0.001			
	Variance				0.018	8.787	0.029
June	75th				0.024	18.637	0.042
	Skewness	-0.003	1.434	0.004			
	Kurtosis	-0.016	2.042	0.000			
	Variance	0.006	5.143	0.075	0.018	9.771	0.014
July	25th	0.013	4.169	0.017	0.022	19.308	0.046
	50th	0.016	5.503	0.014	0.021	21.275	0.049
	75th	0.019	6.972	0.006	0.031	23.844	0.007
	Mean	0.016	6.111	0.012	0.024	21.781	0.023
	Skewness	-0.003	1.235	0.024			
	Kurtosis	-0.009	1.244	0.037	-0.003	-0.369	0.010
	Variance	0.014	6.095	0.029	0.014	11.142	0.009

		T_{min}			T_{max}		
**Month**	**Metric**	**Slope**	**Intercept**	***p***	**Slope**	**Intercept**	***p***
August	25th	0.011	3.402	0.092	0.018	18.762	0.056
	50th	0.015	4.909	0.048	0.022	20.740	0.037
	75th	0.015	6.647	0.029	0.031	23.198	0.004
	Mean	0.015	5.119	0.048	0.023	21.208	0.025
	Skewness	-0.005	1.185	0.001	-0.001	0.404	0.038
	Kurtosis	-0.014	1.402	0.001	-0.003	-0.460	0.003
	Variance				0.009	11.099	0.103
September	50th	0.013	0.954	0.054			
	75th	0.016	2.356	0.036	0.025	18.290	0.096
	Mean	0.011	1.226	0.054			
	Skewness	-0.005	1.087	0.001			
	Kurtosis	-0.013	1.374	0.000	-0.004	-0.543	0.000
	Variance				0.023	8.925	0.000
October	Kurtosis	-0.010	1.428	0.001	-0.003	-0.456	0.011
