# Supplementary material for: The Shifting Climate Portfolio of the Greater Yellowstone Area
Source: PLoS One. 2015 Dec 16;10(12):e0145060. doi: 10.1371/journal.pone.0145060 (PMC4681470; doi:10.1371/journal.pone.0145060)
Supplement: S4 Table — (PDF) [file pone.0145060.s006.pdf]

S4 Table. Management prioritization of species for climate change adaptation action by Yellowstone and Grand Teton national parks staff and partners relative to their Climate Change Sensitivity Database (CCSD) rank and score (http://climatechangesensitivity.org/ accessed on 4/24/2015). Management ranks and scores were developed at a workshop on July 30, 2012 and CCSD ranks and scores are based on expert opinion. Only species that had a management rank and a CCSD rank are shown.

Species	Management Rank	CCSD Rank
Canada lynx	Low (3.06)	High (67)
Moose	Low (3.09)	High (58)
Pika	Low (2.77)	High (63)
Wolverine	Low (3.08)	High (62)
Bald eagle	Medium (3.25)	Medium (28)
Boreal toad	Medium (3.37)	Extremely high (91)
Columbia spotted frog	Medium (3.37)	High (73)
Pronghorn	Medium (3.14)	High (69)
Trumpeter swan	Medium (3.16)	Medium (57)
Bats	High (3.51)	High (68)
Bighorn sheep	High (3.48)	Medium (37)
Elk	High (3.75)	Medium (30)
Gray Wolves	High (4.10)	Low (26)
Grizzly Bear	High (3.94)	Medium (42)
